# Early complications after biliary enteric anastomosis for benign diseases: A retrospective analysis

**DOI:** 10.1186/1471-2482-11-19

**Published:** 2011-08-25

**Authors:** Syed Nabeel Zafar, Muhammad Rizwan Khan, Rushna Raza, Muhammad N Khan, Mahwash Kasi, Ammar Rafiq, Omer H Jamy

**Affiliations:** 1 Dertment of Surgery, Aga Khan University, Stadium Road, Karachi - 74800, Pakistan; 2 Medical College, Aga Khan University, Stadium Road, Karachi - 74800, Pakistan

## Abstract

**Background:**

Biliary-enteric anastomosis (BEA) is a common surgical procedure performed for the management of biliary obstruction or leakage that results from a variety of benign and malignant diseases. Complications following BEA are not rare. We aimed to determine the incidence and the factors associated with early complications occurring after BEA for benign diseases.

**Methods:**

We reviewed the medical records of all patients who underwent BEA for benign diseases at our institution between January 1988 and December 2009. The primary outcome was early post operative complication. Logistic regression analysis was done to identify factors predicting the occurrence of complications.

**Results:**

Records of 79 patients were reviewed. There were 34 (43%) males and 45 (57% females). Majority (53%) had choledocholithiasis with impacted stone or distal stricture, followed by traumatic injury to the biliary system (33%). Thirty-four patients (43%) underwent a hepaticojejunostomy, 19 patients (24%) underwent a choledochojejunostomy, and choledochoduodenostomy was performed in 26 patients (33%). Early complications occurred in 39 (49%) patients - 41% had local complications and 25% had systemic complications. Most frequent complications were wound infection (23%) and bile leak (10%). Four (5%) patients died. On multivariate analysis, low serum albumin level (odds ratio = 16, 95% CI = 1.14-234.6) and higher ASA levels (odds ratio = 7, 95% CI: 1.22-33.34) were the independent factors predicting the early complications following BEA.

**Conclusions:**

Half of the patients who underwent BEA for benign diseases had complications in our population. This high incidence may be explained by the high incidence of hypoalbuminemia and the high-risk group who underwent operation.

## Background

Biliary-enteric anastomosis (BEA) is a common surgical procedure performed for a variety of indications. This includes bypass or reconstruction following resection of malignant or benign biliary obstruction, primary biliary stones, iatrogenic bile duct injury, liver transplantation; and a number of biliary tract problems that are benign but have malignant potential such as primary sclerosing cholangitis, choledochal cyst, and hepatolithiasis. Surgical options for these diverse conditions include hepaticojejunostomy, choledochoenterostomies and cholecystoenterostomies. Biliary-enteric surgery is an essential surgical procedure even for a benign disease etiology [1,2].

Post-operative complications following BEA including anastomotic leak, hemorrhage, wound infection, cholangitis, intra-abdominal abscess/biloma and stricture formation have been reported [3-5]. These complications are sometimes serious enough to warrant a repeat surgery and at times result in serious long-term morbidity. A few studies have looked at factors associated with the development of these complications [6,7]. Patient's age, co-morbid conditions, nutritional status, pre-operative serum bilirubin, associated chronic liver disease, nature and extent of the primary disease and type of anastomosis performed have been proposed to influence the outcome of BEA [7,8]. We aimed to determine the incidence and factors associated with complications following BEA for a group of benign diseases.

## Methods

We retrieved and reviewed the medical records of all patients who underwent BEA for benign diseases at the Aga Khan University Hospital (AKUH), Karachi, Pakistan between January 1988 and December 2009. Since the study spanned over a long period of time, the records with incomplete information were excluded from the study. AKUH is one of the largest private tertiary care hospitals in Pakistan.

Data pertaining to patient demographics, medical history, primary diagnosis, operative procedure, intra-operative details and post-operative outcome were retrieved. The diagnosis of benign disease was identified by pathology records and the hospital coding system. Early complications were defined as any untoward event occurring within 30 days of surgery. Selection of the type of bypass was based on surgeon's preference. All surgeries were performed by consultant general surgeons. There was no established hepatobiliary service at AKUH during the study period.

The primary outcome of early complications was divided into local (wound infection, biliary leak, cholangitis or persistent jaundice, delayed gastric emptying, pancreatic fistula, hemorrhage and pancreatitis) and systemic complications (chest infection, urinary tract infection, venous thromboembolism, pulmonary edema, acute myocardial infarction, renal failure and systemic sepsis). All complications were clumped into a single dichotomous variable called early complication (yes/no), which was used for further analysis as a categorical variable.

Descriptive analysis was performed by calculating proportions for categorical variables and means (with standard deviations) for continuous variables. Univariate and multivariate logistic regression analysis were performed to determine the predictors of early complications. The multivariate model analyzed variables that had a p value of ≤ 0.1 on univariate analysis; in addition to age, gender, American Association of Anesthesiologists (ASA) class, and Charlson Index, which were determined a priori to be clinically significant variables [9].

This research project was a retrospective review of medical records and in accordance with the ethical principles laid forth by the Declaration of Helsinki, retrospective reviews of medical records are exempt from formal ethical review by the ethical review committee of the AKUH. Consent from individual patients was not sought. No identifying information was recorded by the research team.

## Results

A total of 79 adult patients underwent a BEA for a benign pathology. Out of these 79 patients, 45 (57%) were women and 34 (43%) were men. Twenty three (29%) patients had a BMI above 25 Kg/m^2^. Most patients scored 0 (22%) or 1-2 (54%) on the Charlson co-morbidity index, indicating no or mild co-morbidities. The primary diagnosis in most cases (53%) was choledocholithiasis with impacted stone or distal stricture, followed by traumatic injury to the CBD (33%). Seven patients (5.5%) had a benign inflammatory mass resulting in obstruction, while 2 patients each (2.5%) had a choledochal cyst and Mirrizi's syndrome.

The surgical procedures included a hepaticojejunostomy in 34 (43%) patients, choledochojejunostomy in 19 (24%) patients, and choledochoduodenostomy in 26 (33%) patients. Majority of the patients (81%) who underwent a choledochoduodenostomy had a primary diagnosis of choledocholithiasis with or without a distal stricture, while most of the patients (60%) who underwent a hepaticojejunostomy had a diagnosis of traumatic injury to the CBD. Whipple pancreaticoduodenectomy was performed in 4 patients when there was a strong suspicion of malignancy on preoperative assessment. Final histopathology revealed a benign inflammatory mass in 2 cases and benign CBD stricture in 2 cases.

The jejunum was used as a Roux-en-Y loop with end to side anastomosis in 41 (77%) patients and as a loop in 12 (23%). The duodenal anastomoses were fashioned in the usual side to side manner. Majority (65%) of the anastomoses were single layered with interrupted sutures. Eighteen (23%) patients underwent a pre-operative drainage procedure. A stent was used in 28 (35%) patients. Intra-operative bile culture was sent in 77 cases and a positive bacterial growth was documented in 24 (31%) cases.

Early complications occurred in 39 (49%) patients; out of which 18 had more than one complication. Local complication occurred in 32 (41%) patients. The most common local complication was wound infection, followed by biliary leak, as shown in table 1. Systemic complications occurred in 20 (25%) patients. Of the eight patients with biliary leak, one underwent a re-exploration and later on expired of sepsis. The other seven patients were managed either by image guided percutaneous approaches (2 patients) or conservatively (5 patients), and one of them died. Post-operative hemorrhage occurred in two patients - one had an intraperitoneal bleed requiring re-operation, while the other patient developed intra-luminal bleeding and improved on conservative management.

**Table 1:** Frequency of complications after biliary enteric anastomosis for a benign disease

Complications	N (%)
**Local**	
Wound infection	18 (22.8)
Biliary leak	8 (10)
Cholangitis/persistent jaundice	7 (8.9)
Delayed gastric emptying	7 (8.9)
Pancreatic fistula	2 (2.5)
Hemorrhage	2 (2.5)
Pancreatitis	1 (1.3)
**Systemic**	
Chest infection	5 (6.3)
Pulmonary edema	3 (3.8)
Acute myocardial infarction	3 (3.8)
Systemic sepsis	3 (3.8)
Renal Failure	2 (2.5)

**Mortality**	4 (5.1)

The total average duration of hospital stay was 17 (± 12) days with a mean stay of 6 (± 7) days pre-operatively. Postoperatively the mean stay was 12 (± 9) days with a mean stay of 2.2 (± 6) days in the intensive care unit and 3.6 (± 3) days in the special care unit. Four patients (5%) died during hospital stay. Two of these patients underwent Whipple's procedure. Both of these patients had multiple local and systemic complications including bile leak and chest infection, and subsequently died of systemic sepsis with organ failure. The other two patients who died developed wound infection and systemic sepsis; one had concomitant renal failure while the other developed chest infection. A total of 5 patients developed chest infection and 3 of them died, making it the most dreaded systemic complication.

The occurrence of complications was not affected by patient demographic factors, year of surgery, diagnosis or operative technique. The incidence of complications remained similar over the 22-year period. Upon univariate analysis (table 2), patients with albumin levels lower than 3.5 g/dl were 10 times more likely to have a complication (95% CI = 1.10, 98.20). Multivariate analysis (table 3) demonstrated that after adjusting for age, gender, Charlson score and ASA class, patients with an albumin lower than 3.5 g/dl were 16 times more likely to suffer a complication than those with higher albumin levels. Similarly after adjusting for age, gender, Charlson score and albumin level, patients with an ASA class of > 2 were 7 times more likely to suffer a complication than those with a higher ASA class (95% CI: 1.22, 33.34).

**Table 2:** Uni-variate logistic regression analysis of factors associated with early complications in patients undergoing biliary enteric anastomotic surgery for a benign disease

Variable	Category	OR	95% CI
Age (ref: < 50 years)	> 50 years	0.96	0.39, 2.31
Gender (ref: male)	Female	0.78	0.32, 1.90
BMI (ref: ≤25)	> 25 Kg/m^2^	0.86	0.29, 2.53
Albumin (ref: > 3.5)	≤3.5	10.4	1.10, 98.20
Charlson score (re: = 0)	1-2	1.24	0.40, 3.87
	3 and above	2.45	0.64, 9.37
ASA class (ref: 1-2)	> 2	1.30	0.52, 3.33
Primary diagnosis	CBD stones ± stricture	1	-
	Traumatic CBD injury	1.09	0.29, 4.13
	Others	1.4	0.34, 5.77
Surgery	Hepaticojejunostomy	1	-
	Choledochoduoenostomy	0.87	0.31, 2.46
	Choledochojejunostomy	2.26	0.66, 7.76
	Whipple	3.69	0.34, 39.8
Year of Surgery	2003-2009	1	-
	1998-2002	1.17	0.33, 4.12
	1993-1997	1.2	0.31, 4.61
	1988-1992	0.75	0.22, 2.58
Layers of anastomosis (ref: 1 layer)	2 layer	0.95	0.36, 2.55
Stent Placement (ref: yes)	No	1	0.38, 2.62
Sutures (ref: continuous)	Interrupted	1.12	0.44, 2.99
Level of duct (ref: hepatic)	CBD	1.45	0.59, 3.54
Loop (Ref: jejunum)	Duodenum	1.53	0.59, 3.94
Pre -operative drainage (ref: yes)	No	0.46	0.15, 1.43
T Tube placement (ref: yes)	No	0.84	0.28, 2.49
Bile Culture positive (ref: positive)	Negative	1.04	0.40, 2.72

**Table 3:** Multivariate logistic regression analysis of factors associated with early complications in patients undergoing biliary enteric anastomotic surgery for a benign disease

Variable	Category	OR	95% CI
Age (ref: < 50 years)	> 50 years	0.49	0.09, 2.61
Gender (ref: male)	Female	0.52	0.11, 2.61
Albumin (ref: > 3.5)	≤3.5	16.3	1.14, 234.6
ASA class (ref: 1-2)	> 2	6.67	1.22, 33.34
Charlson score (re: = 0)	1-2	0.59	0.07, 5.08
	3 and above	3.5	0.26, 46.3

## Discussion

A high number (49%) of our patients suffered at least one complication after the anastomotic procedure and majority of these were managed conservatively. Complications after biliary-enteric anastomotic procedures have ranged from 3% to 43% in the previous reports [5,10]. However, most of these studies are specific to BEA due to iatrogenic biliary tract injuries. The differing disease etiology, surgery performed and definition of 'complications' make it difficult to make logical comparisons. The largest and most recent of these studies by Sicklick *et al *consisted of 175 patients who underwent biliary reconstruction for the iatrogenic bile duct injury following laparoscopic cholecystectomy found a complication rate of 43% [5]. In another study by Tocchi *et al *consisting of 84 patients undergoing hepaticojejunostomy for benign biliary stricture found a rate of complication of 21% [3]. Even though, there were patients with biliary stricture due to a number of reasons including choledocholithiasis, trauma and choledochal cyst, a hepaticojejunostomy was performed in less than half (43%) of our patients. Also we have included a wider range of conditions in our definition of 'complications', which may have led to higher rates.

Our study included four patients who underwent Whipple pancreaticoduodenectomy due to a strong pre-operative suspicion for a malignant disease. All four of these patients had complications during hospital stay. Our patient population consisted of all patients who underwent BEA with a final diagnosis of a benign disease condition and therefore those four that underwent the Whipple's procedure were included in the analysis. The authors believe that this is a more 'real life' representation as pancreaticoduodenectomies for benign conditions is not a rarity in the literature, and a recent study reviewing 459 pancreaticoduodenectomies for a suspected malignant disease found 11% of them to have a final benign histology [11]. Type of surgery which included a category for 'Whipple's procedure' was not found to be significantly associated with incidence of early complications, however this may be due to lack of power. Furthermore we did perform sensitivity analysis after excluding patients that underwent Whipple's procedure and the results were similar with the incidence of early and local complications being 48% and 39% respectively.

Our study demonstrated a higher incidence of wound infection (23%) as compared to other studies [5]. In both studies by Sicklick et al and Tochi et al, wound infection was the most frequent complication, 8% and 12% respectively [3,5]. Biliary leak occurred in 10% of our patients while it has been reported to occur in 3-8% of patients in previous studies [3,8]. Patients in Pakistan generally present late, are malnourished and thus sicker when compared to patients in the Western world. This is a possible reason for the higher rates of surgical complications in our study. In fact, 87% of our patients had low pre-operative albumin levels (< 3.5 g/dl) and multivariate analysis demonstrated low albumin levels to be an independent predictor of post-operative complications. Previous studies have also shown hypoalbuminemia to influence on in-hospital complications, mortality and long term survival [6,7].

The association between higher ASA classes and the occurrence of complications to the best of our knowledge has not been studied for BEA. ASA class is a measure of functional status of the patient and higher ASA class has been known to lead to a higher rate of complication in other gastrointestinal surgeries [12,13]. ASA class, but not the Charlson score, was shown to be significantly associated to the outcome in our study; we believe this may be reflective of inadequate optimization of patients prior to surgery. To note, none of the other demographic, clinical or operative variables were significantly associated with the outcome. This is similar to other reports [3].

The mortality in our study was 5%. Mortality after BEA for benign biliary stricture has fallen over the years from 10% to 1% in the recent years [3,5,6,8,10]. Half of the deaths in our study occurred in patients who underwent Whipple's pancreaticoduodenectomy; this has not been included in the previous studies. The remaining deaths occurred in patients with multiple complications including renal failure and chest infection. Both of these patients had low pre-operative albumin levels. Due to the small number of deaths (n = 4) we were unable to determine independent factors associated with mortality.

There is no dedicated hepatobiliary service at present at AKUH and all surgeries were performed by the general surgery service. The surgeries were performed by 16 different surgeons over the course of 22 years. Limitations in both financial and human resources make it difficult for institutions in underdeveloped countries to maintain dedicated sub-specialty surgical services. However this may be an important factor contributing to the higher rate of complications as most other reports are from dedicated hepatobiliary centers. Higher volumes of surgery has been associated with better outcomes [14]. A dedicated service offers higher volume to the surgeon and the team. We are currently in the process of developing a number of subspecialty services including pancreatobiliary surgery. It will be interesting to see how outcomes change in the following decades.

The inherent limitation of the retrospective study design made it difficult to account for all potential confounders or study every possible variable that may be related to rate of complications. For example we were unable to retrieve data on time since biliary injury, local ischemia, evolution of surgical technique or other similar factors. However, as we have discussed above, previous studies have also not found an association between other operative, clinical and demographic factors. It would have been interesting to look at factors responsible for specific complications for example wound infection and biliary leak. However in our analysis we did not have enough power to test for these associations. A related limitation is that of the small sample size, we were only able to report on 79 cases; however this is still the largest report of this kind from an underdeveloped country. A larger study looking for associations with specific complications should be conducted.

## Conclusion

BEA for benign conditions has a high rate of post operative complications. Operative technique and type of surgery was not associated with occurrence of complications. Independent factors associated with a high rate of complications are low albumin levels and a higher ASA class. Populations from developing countries may benefit from pre-operative nutritional screening and enhancement.

## Competing interests

The authors declare that they have no competing interests.

## Authors' contributions

MRK and RR were involved in the concept and design of the study. SNZ and MRK were involved data analysis, interpretation and manuscript writing. RR, MNK, AR, AHJ, MK retrieved data from medical records. All authors have reviewed and approved of the final manuscripts.

## Pre-publication history

The pre-publication history for this paper can be accessed here:

http://www.biomedcentral.com/1471-2482/11/19/prepub

## References

[B1] Pitt HA Kaufman SL Coleman J White RI Cameron JL Benign postoperative biliary strictures. Operate or dilate? Ann Surg 1989 210 4 417 425 discussion 426-417 10.1097/00000658-198910000-00001 2802831 PMC1357913

[B2] Nealon WH Urrutia F Long-term follow-up after bilioenteric anastomosis for benign bile duct stricture Ann Surg 1996 223 6 639 645 discussion 645-638 10.1097/00000658-199606000-00002 8645037 PMC1235203

[B3] Tocchi A Mazzoni G Liotta G Lepre L Cassini D Miccini M Late development of bile duct cancer in patients who had biliary-enteric drainage for benign disease: a follow-up study of more than 1,000 patients Ann Surg 2001 234 2 210 214 10.1097/00000658-200108000-00011 11505067 PMC1422008

[B4] Parrilla P Ramirez P Sanchez Bueno F Perez JM Candel MF Muelas MS Robles R Long-term results of choledochoduodenostomy in the treatment of choledocholithiasis: assessment of 225 cases Br J Surg 1991 78 4 470 472 10.1002/bjs.1800780426 2032108

[B5] Sicklick JK Camp MS Lillemoe KD Melton GB Yeo CJ Campbell KA Talamini MA Pitt HA Coleman J Sauter PA Surgical management of bile duct injuries sustained during laparoscopic cholecystectomy: perioperative results in 200 patients Ann Surg 2005 241 5 786 792 discussion 793-785 10.1097/01.sla.0000161029.27410.71 15849514 PMC1357133

[B6] Chapman WC Halevy A Blumgart LH Benjamin IS Postcholecystectomy bile duct strictures. Management and outcome in 130 patients Arch Surg 1995 130 6 597 602 discussion 602-594 7763167 10.1001/archsurg.1995.01430060035007

[B7] Pottakkat B Vijayahari R Prakash A Singh RK Behari A Kumar A Kapoor VK Saxena R Factors predicting failure following high bilio-enteric anastomosis for post-cholecystectomy benign biliary strictures J Gastrointest Surg 14 9 1389 1394 10.1007/s11605-010-1241-8 20589447

[B8] Blankensteijn JD Terpstra OT Early and late results following choledochoduodenostomy and choledochojejunostomy HPB Surg 1990 2 3 151 158 10.1155/1990/62814 2278911 PMC2423582

[B9] Hosmer DW Lemeshow S Applied logistic Regression 2000 2 John Wiley & Sons, Inc

[B10] Laasch HU Martin DF Management of benign biliary strictures Cardiovasc Intervent Radiol 2002 25 6 457 466 10.1007/s00270-002-1888-y 12391514

[B11] Manzia TM Toti L Lenci I Attia M Tariciotti L Bramhall SR Buckels JA Mirza DF Benign disease and unexpected histological findings after pancreaticoduodenectomy: the role of endoscopic ultrasound fine needle aspiration Ann R Coll Surg Engl 92 4 295 301 10.1308/003588410X12628812458374 PMC3025206 20385044

[B12] Garcea G Ganga R Neal CP Ong SL Dennison AR Berry DP Preoperative early warning scores can predict in-hospital mortality and critical care admission following emergency surgery J Surg Res 159 2 729 734 10.1016/j.jss.2008.08.013 19181337

[B13] Story DA Leslie K Myles PS Fink M Poustie SJ Forbes A Yap S Beavis V Kerridge R Complications and mortality in older surgical patients in Australia and New Zealand (the REASON study): a multicentre, prospective, observational study* Anaesthesia 2010 65 10 1022 30 10.1111/j.1365-2044.2010.06478.x 20731639

[B14] Birkmeyer JD Stukel TA Siewers AE Goodney PP Wennberg DE Lucas FL Surgeon volume and operative mortality in the United States N Engl J Med 2003 349 22 2117 2127 10.1056/NEJMsa035205 14645640

